# Regional Variations in Physicochemical Properties and Oil–Solid Interaction Mechanisms of Oily Sludge from Three Chinese Oilfields

**DOI:** 10.3390/toxics14060472

**Published:** 2026-05-27

**Authors:** Yuwei Bao, Jiao Zhao, Yang Liu, Yimin Zhu

**Affiliations:** 1 College of Environmental Science and Engineering, Dalian Maritime University, Dalian 116026, China; 2 Collaborative Innovation Center for Vessel Pollution Monitoring and Control, Dalian Maritime University, Dalian 116026, China

**Keywords:** oily sludge, physicochemical properties, oxidation, oil–solid interaction, oil removal efficiency

## Abstract

Oily sludge is one of the most challenging solid wastes generated during petroleum production and wastewater treatment, posing long-term environmental risks and demanding effective resource-recovery strategies. This study systematically investigated the physicochemical characteristics, compositional differences, and oil–solid interaction mechanisms of oily sludge (OS) from three representative Chinese oilfields, Panjin, Daqing and Xinjiang, through integrated analyses of elemental composition, oil composition, X-ray diffractometer (XRD), Fourier-transform infrared (FT-IR), Gas chromatograph (GC), and Confocal laser scanning microscope (CLSM). The results revealed pronounced regional variations in oxidation degree, hydrocarbon composition, and mineralogy that critically influenced oil occurrence and removal behavior. The Panjin OS sample (PJ-OS) exhibited a high oxidation degree, enriched resins and asphaltenes, and compact film-like oil–solid structures, resulting in the lowest oil mobility and recovery potential. The Daqing OS (DQ-OS) was dominated by light saturates and showed the weakest oil–solid bonding, while the Xinjiang OS (XJ-OS) displayed moderate oxidation and intermediate properties. A novel room-temperature high-speed stirring cleaning method was applied to evaluate oil removal performance under ambient conditions. The residual oil contents after treatment were 4.43% (PJ-OS), 1.65% (DQ-OS), and 1.22% (XJ-OS), corresponding to removal efficiencies of 80.86%, 86.74%, and 90.33%, respectively. The cleaning efficiency was strongly governed by the sludge composition and oxidation state: higher O/C ratios and enrichment of polar heavy fractions enhanced oil–solid adhesion and hindered oil detachment, whereas higher saturate contents and lower oxidation degrees facilitated rapid oil separation. Overall, the findings demonstrate that the treatability of oily sludge is controlled by its intrinsic physicochemical properties. The proposed high-speed stirring technique provides a promising, energy-efficient, and environmentally sustainable approach for oily sludge remediation and resource recovery, offering valuable insights for optimizing treatment parameters and scaling up green petroleum waste management technologies.

## 1. Introduction

Oily sludge (OS) is a primary solid waste generated during the processes of crude oil extraction, transportation, storage, and refining in the petroleum industry, posing significant environmental hazards [1]. A considerable amount of oily sludge is inevitably produced during oil extraction and utilization. According to a survey by the U.S. Environmental Protection Agency, each refinery in the United States generates an average of 3 × 10^4^ tons of oily sludge annually [2]. Estimates from the domestic petrochemical industry suggest that approximately 3 × 10^6^ tons of waste oily sludge are produced each year [3]. In recent years, the global oil refining capacity has continued to expand, resulting in an estimated annual production of over 6 × 10^7^ tons of oily sludge. The cumulative global total of oily sludge has now exceeded 1 × 10^9^ tons [4,5]. Due to its toxicity, oily sludge is classified as hazardous waste, possessing potential carcinogenic and mutagenic properties that pose serious threats to human health and the environment. Nevertheless, due to its high content of petroleum hydrocarbons, it is also viewed as a potential resource for recovery [6]. Given the high oil content and environmental risk characteristics of oily sludge, it represents both environmental challenge and resource opportunity. Therefore, efficient and established methods are necessary for treating such sludge to balance the dual imperatives of environmental protection and resource recovery [7].

The primary treatment methods for oily sludge have traditionally focused on the oil fraction and include pyrolysis, incineration, land farming, chemical treatment, and biodegradation [2]. In contrast, the solid fraction, composed mainly of mineral particles, is typically managed through dewatering, thermal treatment, solidification/stabilization, or controlled land application, depending on the target application or disposal requirements [8]. Several scholars have previously investigated these treatment methods and their effectiveness. For instance, Chen et al. [9] conducted a systematic review of the properties and treatment methods of oily sludge, encompassing studies from various regions both domestically and internationally, and provided a comprehensive analysis of the physicochemical characteristics. Lin. et al. [10] examined the composition and treatment effectiveness of oily sludge in the Daqing oilfield. Furthermore, Hasan et al. [4] analyzed oily sludge treatment technologies across different countries and offered insights into the properties of oily sludge in various regions.

Previous studies indicate that petroleum resources in China are predominantly distributed in the Ordos Basin, Songliao Basin, Bohai Bay Basin, Junggar Basin, and Sichuan Basin. Major oil fields in the country include the Daqing oilfield, Shengli oilfield, Liaohe oilfield, Dagang oilfield, Xinjiang oilfield, and Qinghai oilfield [11]. Notably, the properties and characteristics of oil products vary across these regions. The Panjin oilfield, situated in the lower reaches of the Liaohe River, is characterized by complex geological structures. It ranks as the third-largest oilfield in China and serves as the country’s largest production base for heavy and ultra-heavy oil. Recent exploration and development efforts at the Panjin oilfield primarily focus on the development of heavy oil resources and enhanced oil recovery techniques [12]. The Daqing oilfield, a super-large continental sandstone reservoir, generates oily sludge with a complex composition that includes aged crude oil, asphaltenes, resins, waxes, and various solid impurities. Under typical production and treatment conditions, the sludge contains a relatively high oil content, making complete waste-oil recovery difficult [13]. The Xinjiang oilfield, one of China’s major petroleum production bases situated across extensive desert and Gobi regions, also produces large amounts of oily sludge due to long-term exploitation of both conventional and heavy-oil reservoirs. The oily sludge from this region is characterized by high mineral content, strong weathering effects, and a substantial proportion of heavy and polar fractions, which tend to accumulate in arid soils.

Therefore, this study aims to systematically characterize the physicochemical properties and compositional features of oily sludge samples from three representative regions in China—Panjin, Daqing, and Xinjiang—focusing on the interactions between residual oil and mineral solids within the sludge. Elucidating these oil–solid interactions is crucial for understanding sludge stability and for guiding the development of more effective treatment and disposal strategies. By integrating analytical techniques (elemental analysis, FT-IR, XRD, GC, CLSM, and SARA analysis) with a newly developed room-temperature high-speed stirring cleaning process, this work establishes the correlation between sludge composition, oxidation degree, and oil removal efficiency. The findings provide a theoretical and experimental basis for the design of efficient, low-energy oily sludge treatment and resource recovery technologies.

## 2. Materials and Methods

### 2.1. Samples

Raw oily sludge (OS) samples were collected from primary separation units at three representative oilfields in China—Daqing (DQ-OS), Panjin (PJ-OS), and Xinjiang (XJ-OS). Here, “raw oily sludge” refers to semi-solid waste generated during crude oil extraction, consisting of residual oil, water, and mineral solids (clay, silt, sand), which influence sludge stability and oil–solid interactions. Composite samples were prepared by mixing multiple subsamples from different collection points within each production unit to account for spatial variability. Samples were stored in sealed containers at 4 °C, dried at 70 °C for 18 h to remove moisture and volatiles, and ground to 0.5–1.0 mm particles for subsequent analyses [7]. All experiments were conducted in the Environmental Science and Engineering Laboratory of Dalian Maritime University or the Petroleum Exploration and Development Research Institute Laboratory of Sinopec.

### 2.2. Experimental Procedure

The processing of oily sludge is carried out according to the following experimental procedure [14]. Prior to cleaning, coarse gravel particles (>2 mm), mainly sand and small rock fragments originating from the oil reservoir and produced fluids, were removed by sieving to ensure uniformity of the sludge and focus on the finer oil–solid fraction. In each experiment, 200 g of screened oily sludge was placed into the cleaning vessel, followed by the addition of pentasodium metasilicate (Na_2_SiO_3_·5H_2_O) at a mass ratio of 25:3 (sludge: cleaning agent). Deionized water was then added at a sludge-to-water ratio of 0.5 g mL^−1^. After pre-mixing, the stirring motor was activated, driving the rotor to rotate at high speed within a fixed cylindrical chamber. During operation, the sludge slurry was drawn from the bottom of the chamber and ejected through circular outlet holes at high velocity, forming a continuous circulation loop that enabled strong turbulence and effective mechanical cleaning. The separated crude oil floated to the liquid surface and was removed by skimming, while the remaining mixture was dehydrated by centrifugal separation. The residual oil content of the cleaned sludge was determined according to the Chinese national standard HJ 637–2012: Soil and Sediment—Determination of Petroleum Hydrocarbons by Infrared Spectrophotometry and Determination of Petroleum in Soil by Infrared Photometry (Draft for Comments) [15]. Each test was conducted in triplicate, and the mean values were reported to ensure reproducibility.

### 2.3. Characterization Analysis

The oily sludge was comprehensively analyzed for its ultimate contents of carbon (C), hydrogen (H), oxygen (O), nitrogen (N), and sulfur (S) by Thermo Scientific Flash 2000 Elemental Analyzer (Waltham, MA, USA). The functional groups in the oily sludge were characterized by Nicolet 6700 Fourier-transform infrared (FT-IR) spectrometer (Green Bay, WI, USA) over a spectral range of 4000–400 cm^−1^ with a resolution of 4 cm^−1^. Additionally, the mineral composition of the oily sludge was analyzed by Bruker D8 Advance X-ray diffractometer (XRD) (Berlin, Germany), and the relative proportions of minerals were estimated based on the areas of the major peaks.

### 2.4. GC and SARA Analyses

Saturated and aromatic fractions were analyzed using a Thermo Trace DSQ gas chromatograph–mass spectrometer (GC, DSQ Dual Stage Quadrupole) (Waltham, MA, USA). The system was equipped with an HP-5ms capillary column (60 m × 0.25 mm × 0.25 µm). The oven temperature was initially set at 60 °C for 1 min, ramped to 260 °C at 6 °C/min, then to 320 °C at 1.5 °C/min and held for 20 min. Helium was used as the carrier gas at 1.0 mL/min. The mass spectrometer was operated in electron ionization (EI) mode at 70 eV, with an ion source temperature of 250 °C, a transfer line temperature of 280 °C, and a mass scan range of m/z 50–500 [16].

The SARA (saturates, aromatics, resins, and asphaltenes) fractionation of oily sludge samples was performed following the SY/T 5119-2016 standard [17]. Initially, the sludge samples were subjected to Soxhlet extraction with chloroform to obtain the total chloroform-soluble asphalt, denoted as “A.” Asphaltenes were then precipitated by adding excess n-hexane, filtered, washed with n-hexane to remove soluble impurities, and dried to constant weight. The n-hexane–soluble portion was separated on a silica–alumina column using sequential elution with solvents of increasing polarity: n-hexane for saturates, toluene for aromatics, and a mixture of toluene and methanol (95:5, *v*/*v*) for resins. Elution was carried out with solvents of progressively increasing polarity to sequentially recover the saturate, aromatic, and resin fractions. Each collected fraction was evaporated to dryness under controlled conditions until a constant mass was achieved, and the mass percentages of the four SARA components were calculated based on their respective weights [18,19].

### 2.5. CLSM and Fluorescence Analyses

In this study, CLSM analysis was performed by LEICA SP5 II confocal laser scanning microscope (Leica Microsystems Office, Shanghai, China). Prior to observation, the samples were cryo-sectioned into thin slices of approximately 300–500 μm to ensure optimal laser penetration. Fluorescence intensity and distribution were analyzed using LAS AF software (LAS AF 3.2), and images were reconstructed in 3D mode to visualize oil–solid spatial relationships. The microscope was equipped with a universal spectral fluorescence detector and an objective lens shared with the scanning laser. To induce autofluorescence, a traditional high-pressure mercury lamp was used, with an excitation wavelength range of 200–400 nm (UV light). In the actual analysis, a laser with an excitation wavelength of 488 nm (blue light) was selected as the light source to generate two-dimensional (2D) images of the samples. The three-dimensional (3D) structure of the samples was reconstructed by imaging thin sections at different depths and adjusting the focal depth to acquire images at various levels [20].

## 3. Results and Discussion

### 3.1. Physicochemical Characteristics

The PJ-OS sample exhibited a black appearance, with residual oil encapsulating fine soil particles and other mineral solids, primarily clay and silt naturally present in the reservoir. These fine solids contribute to uneven oil distribution and are central to oil–solid interactions. The DQ-OS contained coarser sand and gravel from the reservoir, increasing density and moderating oil distribution, but they were excluded from detailed analysis as the study focuses on the finer fraction relevant to sludge stability and treatment efficiency. The XJ-OS displayed irregular morphology with a dark brown surface and black interior; coarse gravel and sand were noted but similarly excluded from in-depth analyses. Overall, the mineral solids observed in all samples are natural components from the crude oil reservoirs, shaping sludge properties and influencing oil retention, rheology, and cleaning performance.

Elemental analysis of the three oily sludge samples, PJ-OS, DQ-OS, and XJ-OS, revealed significant regional differences in carbon (C), hydrogen (H), and oxygen (O) contents, as well as in their atomic ratios (Table 1) [21]. The carbon content ranged from 53% to 57%, indicating that all samples are rich in organic matter and exhibit typical characteristics of petroleum-derived contaminants [22]. Hydrogen contents were between 7% and 9%, corresponding to hydrogen-to-carbon atomic ratios (H/C) of 1.67–1.89, suggesting the presence of hydrogen-rich components in all samples [22]. Generally, a higher H/C ratio reflects a greater proportion of aliphatic structures and higher hydrogen enrichment, whereas a lower ratio indicates a higher degree of aromatization or oxidation [23]. Among the samples, the PJ-OS exhibited the highest H/C ratio (1.89), implying the retention of more hydrogen-enriched organic structures. The DQ-OS and XJ-OS samples showed slightly lower H/C ratios (1.67 and 1.85, respectively), suggesting relatively more mature organic compositions [24]. Oxygen contents varied considerably (15.65–21.00%), corresponding to oxygen-to-carbon atomic ratios (O/C) of 0.21–0.28. The PJ-OS exhibited the highest O/C ratio (0.28), indicating a stronger degree of oxidation and a higher proportion of polar functional groups [25]. The DQ-OS (0.24) and XJ-OS (0.21) samples had comparatively lower O/C ratios, suggesting better preservation of organic matter [23,26]. Nitrogen contents were relatively low (1.11–1.40%), and no significant sulfur content was detected, implying that sulfur-containing compounds were scarce or had been oxidized and removed during weathering [27]. Overall, the XJ-OS, characterized by a low O/C ratio and a relatively high H/C ratio, shows better preservation of hydrocarbons compared with the other samples. The DQ-OS exhibits intermediate characteristics, while the PJ-OS, despite its high O/C ratio, displays the strongest oxidation and the highest proportion of polar components. Consequently, enhanced oil–mineral interactions reduce oil mobility, leading to lower recovery efficiency and increased difficulty in sludge cleaning. The oil content and SARA (saturates, aromatics, resins, and asphaltenes) composition of OS samples from Panjin, Daqing, and Xinjiang exhibited distinct regional variations, reflecting differences in hydrocarbon composition, oxidation degree, and aging characteristics (Table 2). The oil content of the samples ranged from 12% to 23%, with the PJ-OS showing the highest value (23.14%), followed by the DQ-OS (12.44%) and XJ-OS (12.61%). The relatively high oil content of the PJ-OS, together with its elevated proportions of resins (18.11%) and asphaltenes (10.15%), indicates that this sample has undergone extensive oxidation, resulting in significant enrichment of polar heavy fractions. This leads to increased sludge viscosity and stronger binding between oil and mineral particles, ultimately reducing oil recovery efficiency [28]. In contrast, the DQ-OS was dominated by saturates (58.45%), with relatively low contents of resins (9.16%) and asphaltenes (3.25%), suggesting a fresher hydrocarbon composition, weaker oxidation, and higher oil mobility—conditions more favorable for oil separation and recovery [23,29]. The XJ-OS comprised 51.18% saturates, 32.35% aromatics, 11.59% resins, and 4.88% asphaltenes, indicating a moderate degree of oxidation and aging. Although heavy fractions were partially enriched, a considerable proportion of light hydrocarbons was still preserved [30,31,32]. Overall, the SARA analysis results are consistent with the elemental composition characteristics discussed earlier, revealing a clear compositional gradient among the samples: the PJ-OS exhibits the highest oxidation degree and the poorest treatability; the DQ-OS possesses the best hydrocarbon quality and greatest recovery potential; and the XJ-OS shows intermediate characteristics.

**Figure 1:**
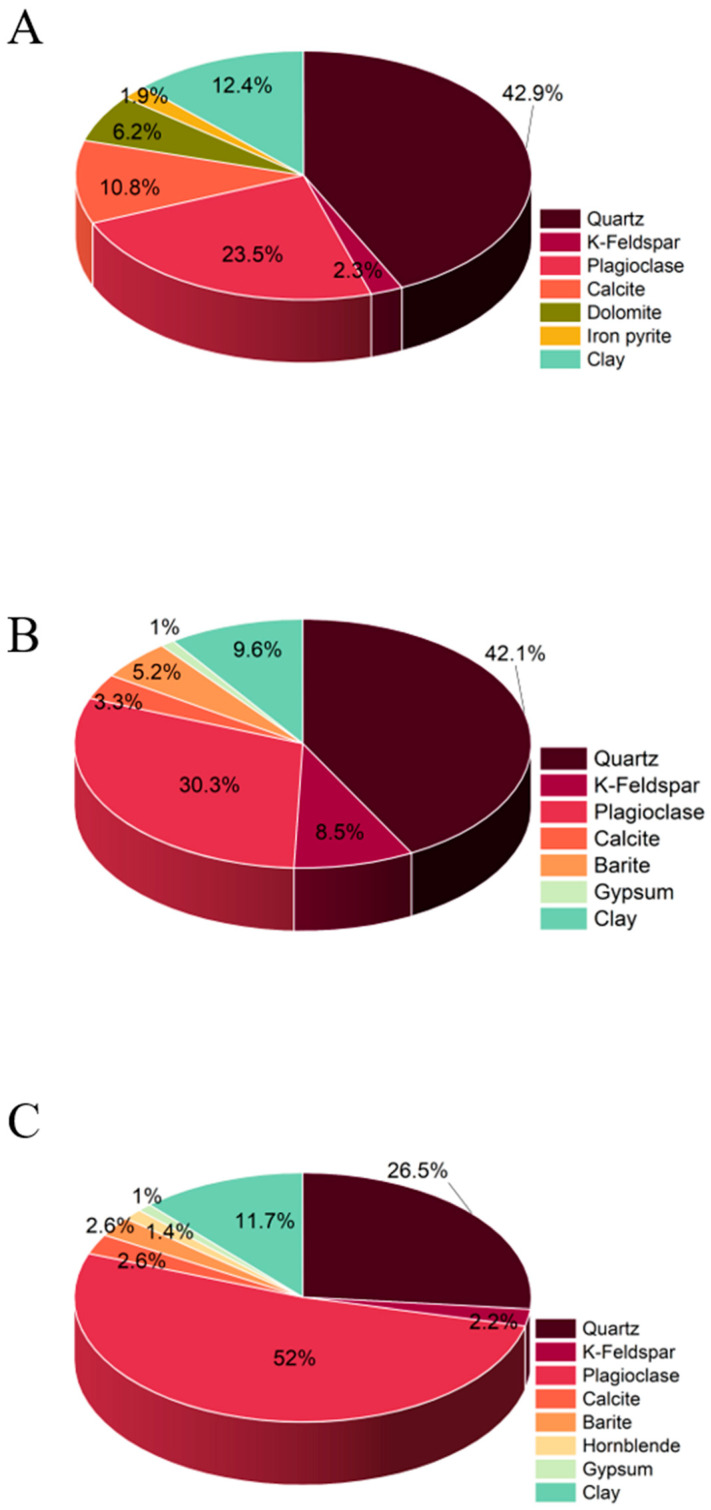
Whole rock mineral composition of (**A**) PJ-OS sample, (**B**) DQ-OS sample, and (**C**) XJ-OS sample.

Whole-rock mineralogical analysis was performed to characterize the inorganic constituents of the oily sludge, as shown in Figure 1. The dominant mineral phases of oily sludge from the three regions are quartz and feldspar-type silicates, accounting for approximately 65–80% of the total composition, indicating that the solid matrix primarily originates from reservoir sandstone particles generated during oil extraction processes [24,33,34]. Significant variations are observed in plagioclase content: the XJ-OS contains as much as 52.0%, which is considerably higher than that in PJ-OS (23.5%) and DQ-OS (30.3%). In contrast, the PJ-OS exhibits a relatively lower plagioclase fraction but a higher proportion of carbonate minerals such as calcite and dolomite. The PJ-OS is characterized by abundant quartz (42.9%) and carbonates (calcite 10.8%, dolomite 6.2%), suggesting a pronounced influence of carbonate cementation. The presence of Ca^2+^ and Mg^2+^ bridges facilitates complexation or adsorption with polar functional groups in the oil phase, resulting in strong oil–solid adhesion and greater difficulty in separation [35]. The detection of pyrite (1.9%) further indicates a locally reducing environment, which favors organic matter preservation and oily sludge formation [36]. The DQ-OS contains a high proportion of silicate detritus, dominated by quartz (42.1%) and feldspars (K-feldspar 8.5%, plagioclase 30.3%), along with barite (5.2%) and minor gypsum (1.0%). The XJ-OS is dominated by plagioclase (52.0%) and contains only 26.5% quartz, along with detectable hornblende (1.4%) and small amounts of barite and gypsum. This composition indicates a parent rock enriched in ferromagnesian silicate minerals. Such minerals possess dense crystal structures and abundant surface hydroxyl groups, which enhance hydrophilicity; therefore, their higher density can facilitate the rapid detachment and sedimentation of oil droplets during the washing process [37].

The total clay mineral content of the three samples ranges from 9.6% to 12.4%, mainly consisting of illite, kaolinite, and montmorillonite [38]. Clay minerals have a large specific surface area and a high density of negative charges, enabling strong adsorption of polar components and surfactants from crude oil, thereby forming a stable emulsified film that contributes to the structural stability of oily sludge [38]. The PJ-OS exhibits the highest clay content (12.4%), consistent with its higher initial oil content, indicating that clay minerals play a crucial role in oil–solid association and emulsion stability.

**Figure 2:**
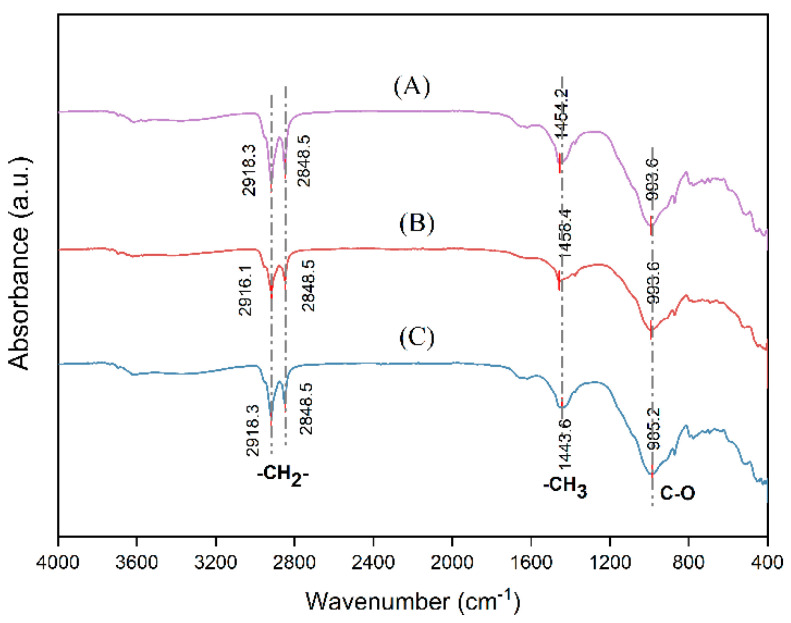
FT-IR spectra of (**A**) PJ-OS, (**B**) DQ-OS, and (**C**) XJ-OS.

The FT-IR spectra of oily sludge samples, PJ-OS (A), DQ-OS (B), and XJ-OS (C), are presented in Figure 2. All spectra display similar characteristic absorption bands, indicating that the organic matter in the sludge mainly originates from petroleum hydrocarbons, while differences in band intensity and slight peak shifts reflect compositional and structural variations among regions [39]. The strong absorption peaks observed at 2918–2916 cm^−1^ and 2848 cm^−1^ correspond to the asymmetric and symmetric stretching vibrations of –CH_2_– groups in long-chain aliphatic hydrocarbons, respectively. These bands are typical of saturated C–H bonds in paraffinic and alicyclic compounds, confirming that aliphatic components dominate the organic fractions of all three samples [40]. The band near 1458–1443 cm^−1^ is attributed to the bending vibration of –CH_3_ groups, further supporting the predominance of alkane-type hydrocarbons. The relatively higher intensity of these C–H vibrations in spectra (A) and (B) compared with (C) suggests that the PJ-OS and DQ-OS contain a greater proportion of long-chain hydrocarbons and waxy substances, whereas the XJ-OS is relatively depleted in such compounds [41]. A weak absorption band at 993–965 cm^−1^ is assigned to C–O stretching or Si–O–Si bending vibrations, which may originate from alcohols, ethers, or silicate minerals within the inorganic matrix. The presence of this band in all samples implies partial oxidation or adsorption of oxygen-containing functional groups on mineral surfaces. The XJ-OS sample (C) shows a slight blue shift in this peak (965 cm^−1^), suggesting stronger mineral–organic interactions, consistent with its higher plagioclase and ferromagnesian silicate contents revealed by XRD [42]. Overall, the FT-IR spectra confirm that the organic fractions of all three OS samples are dominated by aliphatic hydrocarbons with minor oxygenated functionalities. The intensity differences and peak shifts among regions reflect variations in oil composition, degree of weathering, and mineral binding strength. Specifically, the PJ-OS exhibits the strongest C–H stretching signals, indicating heavier paraffinic fractions and higher residual oil content; the DQ-OS shows intermediate characteristics; while the XJ-OS, showing relatively stronger oxygen-containing functional groups, may have undergone greater oxidation or adsorption onto silicate surfaces.

### 3.2. Analysis of the Oil Phase with GC

The soluble compounds in the OS samples were comprehensively characterized using gas chromatography (GC). Normal alkanes (n-alkanes) and aromatic hydrocarbons were identified by spectral library matching, confirming the presence of medium- and long-chain n-alkanes as well as mono- and polycyclic aromatics in all three samples.

**Figure 3:**
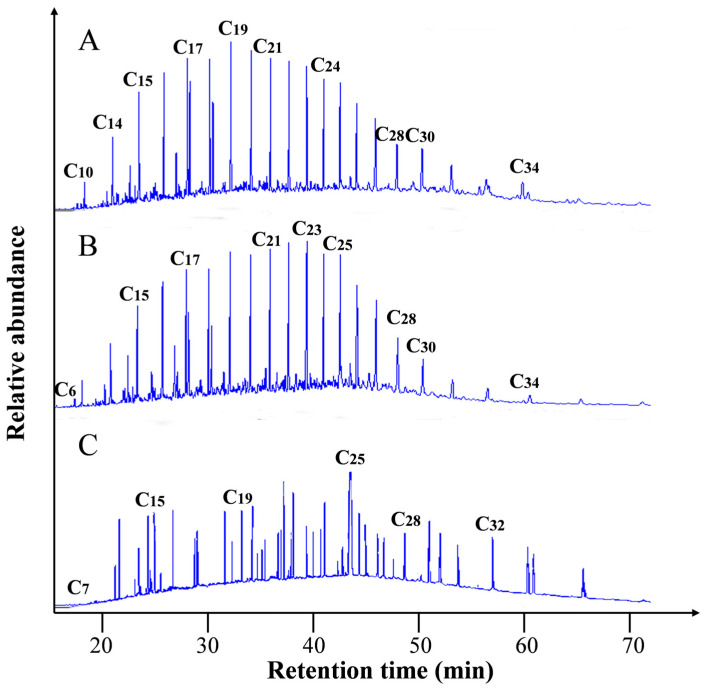
GC chromatograms of (**A**) PJ-OS sample, (**B**) DQ-OS sample, and (**C**) XJ-OS sample.

In the PJ-OS (Figure 3A), the chromatographic peaks are relatively concentrated within the range of nC_13_ to nC_29_, showing a regular and relatively smooth distribution. The signals of the mid- to high-carbon components (C_17_–C_23_) are particularly strong, indicating that the hydrocarbons are dominated by medium-chain aliphatic compounds [43]. The overall peak envelope exhibits a certain degree of symmetry, suggesting a relatively uniform aliphatic hydrocarbon source, with partial volatilization or oxidation of light components [44]. These observations are consistent with the higher O/C ratio and enrichment of resin and asphaltene fractions in the PJ-OS, reflecting pronounced oxidative aging and the partial loss of light hydrocarbons, accompanied by relative accumulation of polar heavy fractions. The DQ-OS (Figure 3B) displays a broader distribution of n-alkane peaks, covering approximately nC_11_ to nC_31_. The peaks are well-defined and exhibit high intensities, forming a typical “bell-shaped” distribution—where the mid-carbon range (C_16_–C_20_) shows the highest peak values, while both the low- and high-carbon ends are relatively suppressed. This pattern indicates that the DQ-OS retains abundant light and medium molecular weight hydrocarbons, with moderate thermal maturity and a low degree of oxidation [45]. These characteristics are consistent with the SARA results, where saturates account for the highest proportion (58.45%), suggesting high oil activity and enhanced recoverability compared with the Panjin OS. The XJ-OS (Figure 3C) exhibits generally lower overall peak intensities; however, the reduced signal strength primarily results from vertical scale adjustment rather than a substantial decrease in actual hydrocarbon content. The peaks span a relatively wide range (approximately nC_13_ to nC_33_), with a higher proportion of medium- to long-chain components (beyond C_20_) and a flatter distribution. This indicates partial loss of light hydrocarbons and enrichment of heavier fractions [46]. Combined with the previously discussed H/C ratios and SARA results, these findings suggest that the organic matter in the XJ-OS is relatively well preserved but has undergone slight oxidation, with residual oil mainly composed of medium- and long-chain hydrocarbons.

Although absolute hydrocarbon contents were not quantified due to the focus on distribution trends and oil–solid interactions, the GC chromatograms reliably reveal the relative abundance and molecular range of n-alkanes and aromatics in each sample. Combined with SARA fractionation and H/C ratio analyses, these observations support interpretations of thermal maturity, partial oxidation, and the role of oil–solid interactions in determining sludge physicochemical characteristics.

### 3.3. Microscopic Oil Analysis with CLSM

**Figure 4:**
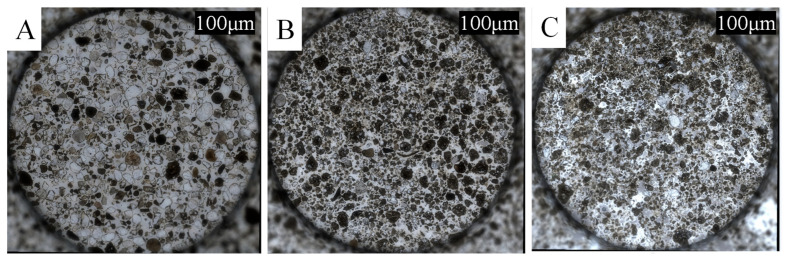
Large-field imaging of OS samples by confocal laser scanning microscopy (CLSM). (**A**) PJ-OS sample. (**B**) DQ-OS sample. (**C**) XJ-OS sample.

**Figure 5:**
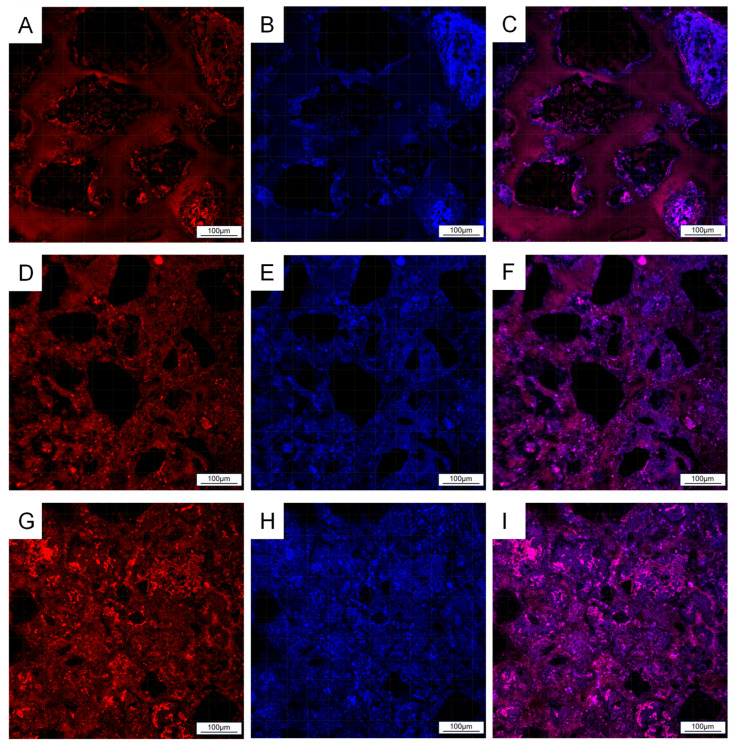
Distribution of light components, and heavy components in the PJ-OS sample (**A**–**C**), DQ-OS sample (**D**–**F**), and the XJ-OS sample (**G**–**I**). (**A**,**D**,**G**) light component. (**B**,**E**,**H**) heavy component. (**C**,**F**,**I**) overlapping images of light and heavy components.

**Figure 6:**
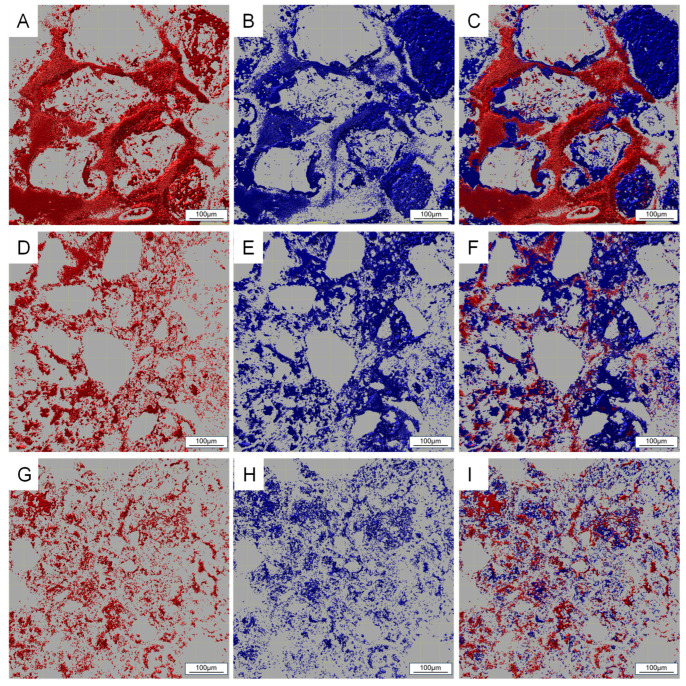
Microphotographs showing the 3D reconstruction of samples based on the CLSM for PJ-OS sample (**A**–**C**), DQ-OS sample (**D**–**F**), and XJ-OS sample (**G**–**I**). (**A**,**D**,**G**) light component. (**B**,**E**,**H**) heavy component. (**C**,**F**,**I**) overlapping images of light and heavy components.

Conventional fluorescence microscopy is inadequate for analyzing the complex distribution of organic matter in oily sludge due to its limited spatial resolution and the image blurring caused by the high content of fluorescent components in sediments [47]. In contrast, confocal laser scanning microscopy (CLSM), offering nearly tenfold higher resolution than conventional optical microscopy, enables clear imaging of cryosectioned samples less than 0.5 mm thick [48]. This technique effectively minimizes interference from particle overlap and background fluorescence while preserving the in situ spatial relationships among oil, water, and mineral phases, enabling nondestructive microstructural characterization [49]. In fluorescence mode, CLSM was employed to investigate oily sludge samples from the three oilfield regions, providing clear, high-resolution optical sections without image blurring (Figure 4). CLSM allowed detailed observation of organic–mineral associations and differentiation of hydrocarbon fractions based on their fluorescence responses, which are closely related to molecular composition and viscosity. Specifically, light hydrocarbon fractions—primarily saturates and low-molecular-weight aromatics—exhibited stronger and more uniform fluorescence signals, whereas heavy fractions—dominated by resins and asphaltenes—displayed weaker, heterogeneous fluorescence due to their higher polarity and viscosity [49]. Figure 5 and Figure 6 present the spatial distribution and three-dimensional reconstruction of light and heavy hydrocarbon fractions in oily sludge samples from Panjin (PJ-OS), Daqing (DQ-OS), and Xinjiang (XJ-OS). The CLSM images were acquired using dual-channel fluorescence detection, in which red fluorescence represents light SARA components (saturates and aromatics), while blue fluorescence corresponds to heavy SARA components (resins and asphaltenes). The merged images (C, F, I) provide a direct visualization of the intermixing and phase continuity between these fractions [49]. In the PJ-OS sample (Figure 5A–C), light components dominate the organic phase and are distributed continuously along the mineral particle surfaces. The heavy components appear as localized aggregates, indicating that the sludge matrix contains abundant residual oil with a relatively low degree of oxidation or weathering [50]. The continuous oil films observed in the 3D reconstruction (Figure 6A–C) further demonstrate the strong oil–solid adhesion and compact structure, consistent with the high residual oil content determined by extraction analysis. In contrast, the DQ-OS sample (Figure 5D–F) exhibits a more dispersed distribution of both light and heavy components. The heavy fraction is concentrated within intergranular pores, whereas the light hydrocarbons form a thinner coating around mineral particles. The reconstructed microstructure (Figure 6D–F) reveals a more open pore network and reduced continuity of oil films, suggesting that the sludge from Daqing has experienced moderate degradation and partial desorption of hydrocarbons during production or storage [49]. The XJ-OS sample (Figure 5G–I) shows the weakest fluorescence intensity, indicating a lower overall hydrocarbon content. Both light and heavy components are finely dispersed and discontinuous, with the heavy components primarily confined to small isolated clusters. The corresponding 3D reconstruction (Figure 6G–I) confirms a highly fragmented and porous microstructure, implying efficient natural aging and oxidation processes, which reduce the viscosity and interfacial adhesion of the residual oil [50].

Overall, the CLSM results reveal a clear regional trend: the Panjin sample is characterized by compact oil films and strong binding between hydrocarbons and solids; the Daqing sample exhibits partial oil detachment and structural loosening; while the Xinjiang sample displays extensive degradation and mineral exposure.

### 3.4. Cleaning Efficiency and Implications

To verify the oil removal efficiency for oily sludge of different physicochemical properties under a given cleaning method, the OS samples from Panjin (PJ-OS), Daqing (DQ-OS), and Xinjiang (XJ-OS) were treated using the room-temperature high-speed stirring cleaning technique [13]. After cleaning, the residual oil contents of the treated sludge samples were 4.43% (PJ-OS), 1.65% (DQ-OS), and 1.22% (XJ-OS), corresponding to oil removal efficiencies of 80.86%, 86.74%, and 90.33%, respectively. Among them, PJ-OS exhibited the lowest removal efficiency (80.86%), primarily due to its higher oxidation degree and the enrichment of polar heavy fractions such as resins and asphaltenes. In contrast, the DQ-OS sample showed a lower residual oil content (1.65%) and a higher removal efficiency (86.74%). CLSM imaging revealed that light hydrocarbons in DQ-OS were uniformly distributed and exhibited minimal overlap with heavy fractions, forming mobile, easily detachable droplets. This morphology indicates weaker oil–solid interactions and enhanced oil mobility, thereby facilitating efficient oil separation during high-speed stirring. The XJ-OS sample achieved the highest overall removal efficiency (90.33%). Although moderately oxidized, its loose microstructure and relatively high clay mineral content promoted intense shear action and increased particle–fluid contact, which enhanced the mechanical stripping of oil films. The medium viscosity and dispersed droplet morphology observed in CLSM images further corroborate its superior cleaning performance. The treatment results of oily sludge samples from different regions using the room-temperature high-speed stirring cleaning technique demonstrate that this method achieves satisfactory oil removal performance across sludge with diverse crude oil compositions and physicochemical characteristics. However, the cleaning efficiency is strongly governed by the compositional complexity and microstructural features of the sludge. The PJ-OS sample, enriched in polar heavy fractions such as resins and asphaltenes, exhibited strong oil–solid interactions that impeded the detachment of bound oil during cleaning. In contrast, the DQ-OS and XJ-OS samples, characterized by higher contents of light components, more uniform oil droplet distributions, and looser aggregate structures, displayed enhanced oil mobility and superior separation efficiency. These findings indicate that the room-temperature high-speed stirring method possesses broad applicability and operational feasibility, yet further optimization should be adapted to the intrinsic physicochemical properties of oily sludge from different sources.

Future studies should focus on three main aspects. First, refining key process parameters—including shear intensity, cleaning agent formulation, and multi-stage operation—may further enhance the treatment efficiency of highly oxidized or heavy-fraction-enriched sludge. Second, integrating advanced multi-scale characterization techniques (e.g., in-situ infrared spectroscopy, low-field nuclear magnetic resonance, and confocal microscopy) is essential for elucidating the mechanisms that govern oil–solid interfacial evolution during cleaning. Third, coupling this technique with other synergistic or enhanced treatment processes could facilitate the development of an efficient, low-energy, and sustainable system for oily sludge cleaning and resource recovery. Overall, this study provides a scientific basis for understanding the regional adaptability of the room-temperature high-speed stirring cleaning process and offers valuable insights into the advancement of next-generation oily sludge reduction and valorization technologies.

## 4. Conclusions

Comprehensive physicochemical and microstructural analyses of oily sludge samples from Panjin, Daqing, and Xinjiang revealed distinct regional differences in composition, oxidation degree, and oil–solid interaction mechanisms. The PJ-OS sample exhibited a high oxidation level, enrichment of polar heavy components (resins and asphaltenes), and dense oil–solid film structures, resulting in strong adhesion and low oil recoverability. The DQ-OS sample, dominated by light saturates and low oxygen content, displayed the most favorable oil mobility and weakest oil–solid bonding, while the XJ-OS showed moderate oxidation and intermediate behavior. Application of the room-temperature high-speed stirring cleaning process achieved efficient oil removal, with residual oil contents of 4.43%, 1.65%, and 1.22% for PJ-OS, DQ-OS, and XJ-OS, respectively. The cleaning efficiency exhibited a strong correlation with oxidation degree and compositional characteristics: higher O/C ratios and enrichment of polar fractions increased oil–solid adhesion and reduced removal efficiency, whereas a higher proportion of saturates and lower oxidation levels enhanced oil detachment and recovery.

In conclusion, the treatability of oily sludge is fundamentally determined by its intrinsic chemical composition and oxidation state. The proposed high-speed stirring method offers a low-energy, non-thermal, and environmentally sustainable solution for oily sludge remediation and petroleum waste valorization. The outcomes of this study provide theoretical and technical guidance for the development of scalable, green, and efficient treatment technologies for petroleum-based solid wastes.

## Figures and Tables

**Table 1:** Organic elemental analysis results.

Samples	Analysis Result						
	N (%)	C (%)	H (%)	S (%)	O (%)	H/C	O/C
PJ-OS	1.20	57.00	9.00	/	21.00	1.89	0.28
DQ-OS	1.11	53.81	7.47	/	17.19	1.67	0.24
XJ-OS	1.40	55.97	8.61	/	15.65	1.85	0.21

**Table 2:** Oil concentration and SARA composition of OS samples.

Samples	Oil Content (%)	SARA Composition			
		Saturates (%)	Aromatics (%)	Resins (%)	Asphaltenes (%)
PJ-OS	23.14	35.76	35.98	18.11	10.15
DQ-OS	12.44	58.45	29.14	9.16	3.25
XJ-OS	12.61	51.18	32.35	11.59	4.88

## Data Availability

The original contributions presented in this study are included in the article. Further inquiries can be directed to the corresponding author.
